# A Phase IB Trial of the PI3K Inhibitor Alpelisib and Weekly Cisplatin in Patients with Solid Tumor Malignancies

**DOI:** 10.1158/2767-9764.CRC-22-0028

**Published:** 2022-07-01

**Authors:** Erica S. Tsang, Rahul R. Aggarwal, Mallika S. Dhawan, Emily K. Bergsland, Edwin A. Alvarez, Susan Calabrese, Romain Pacaud, Jose Garcia, Delaire Fattah, Scott Thomas, Jennifer Grabowsky, Mark M. Moasser, Pamela N. Munster

**Affiliations:** Division of Hematology and Oncology, University of California San Francisco, San Francisco, California.

## Abstract

The PI3K pathway may be a potential mechanism to overcome cisplatin resistance. We conducted a phase Ib trial of alpelisib and cisplatin for patients with solid tumor malignancies with planned dose expansion in HPV-associated tumors. The primary objective was to determine the MTD and recommended phase II dose. Two different weekly doses of cisplatin (30 and 35 mg/m^2^) were evaluated with escalating doses of alpelisib, administered daily during a 21-day treatment cycle. Twenty-three patients were enrolled: 91% received >3 prior regimens with median of 4 (range 1–10), and 78% progressed on prior platinum. The MTD was alpelisib 250 mg daily with weekly cisplatin 30 mg/m^2^. There were 3 DLTs: all grade 4 hyperglycemia. Frequent treatment-related adverse events of any grade included fatigue (52%), diarrhea (39%), nausea (38%), hyperglycemia (30%), anemia (22%), and nephropathy (17%). Hyperglycemia was linked to baseline hemoglobin A1C, but not body mass index. Twelve patients discontinued treatment for toxicity (*n* = 9 during cycle 1) and 11 discontinued for progression. Of 14 evaluable patients who received at least one treatment cycle, 4 (29%) patients demonstrated partial response, and 7 had stable disease for a disease control rate of 79%. The median PFS measured 4.3 months (95% CI, 1.6–4.5). No difference in PFS was observed between *PIK3CA*-mutated and wild-type tumors. While the combination of alpelisib and cisplatin demonstrated preliminary evidence of activity despite platinum resistance, toxicities hindered prolonged treatment. Prospective studies are planned using carboplatin and alpelisib to improve toxicity and tolerability.

**Significance:**

The PI3K inhibitor alpelisib has limited activity alone, but there is interest in combinations in platinum-resistant tumors. In this phase Ib study of alpelisib with cisplatin, the objective response rate measured 29% but adverse events limited dose intensity. These promising results provide rationale for studying combinations with better tolerated platinum agents.

## Introduction

A significant number of tumors demonstrate mutations in the PI3K pathway and substantial efforts have been made to target this pathway, testing PI3K and downstream modulators (mTOR and AKT inhibitors) in multiple tumor settings. However, to date, only a few agents targeting this pathway have been approved. Most notably, two inhibitors of this pathway have been approved in hormone-sensitive breast cancer, the mTOR inhibitor everolimus in mutation agnostic tumors and the selective inhibitor of the alpha isoform of PI3K, alpelisib, in tumors with PI3K alterations, both in combination with hormonal therapy (1, 2). Alpelisib is currently being studied in other diseases and multiple other combinations, but single-agent activity and combination effects have been limited. The relevance and extent of tumors with perturbed PI3K pathways continues to drive the quest in finding rational combinations in tumors dependent on the PI3K pathways, or where an activated PI3K pathway may contribute to chemotherapy resistance.

Cisplatin chemotherapy remains the mainstay of treatment for patients with advanced human papillomavirus (HPV)-associated tumors, from head and neck squamous cell carcinoma to cervical cancer (3, 4). Despite established efficacy in the first-line setting, subsequent relapse is common and repeat cisplatin administration in the second-line setting is often much less successful and too toxic. The PI3K pathway has been explored as a potential mechanism to overcome cisplatin resistance. Preclinical studies in cisplatin-resistant ovarian cancer cell lines demonstrated that PI3K pathway inhibition sensitized cells to cisplatin-induced apoptosis (5). Other *in vitro* models of squamous cell cancer also showed a similar synergistic effect, with PI3K inhibition enhancing cisplatin efficacy. Adding to this rationale, PI3K pathway activation and HPV-associated carcinogenesis are closely related, with specific oncoproteins playing a direct role in pathway activation (6). Somatic alterations involving PI3K pathway activation have been reported in 45%–60% of HPV-associated oropharyngeal squamous cell carcinoma and in 15% of cervical cancers (7, 8).

While early phase trials have demonstrated safety and efficacy of alpelisib with and without cetuximab in patients with advanced head and neck cancers, emerging data points to efficacy in head and neck cancer favoring cisplatin over cetuximab (9). However, alpelisib has not been combined with the standard-of-care cisplatin in this population (10, 11). Given the strong justification for combining PI3K inhibition with cisplatin in HPV-driven tumors, we conducted a phase Ib trial of combination alpelisib with cisplatin for patients with solid tumor malignancies and HPV-associated malignancies in the dose expansion cohort.

## Materials and Methods

### Patient Population

Patients with advanced solid tumor malignancies were enrolled in the dose escalation cohort, and those with HPV-associated tumors were eligible for the dose expansion cohort. Eligible patients were required to have an Eastern Cooperative Oncology Group performance status of ≤1, absolute neutrophil count ≥1.0 × 10^9^/L, platelet count ≥100 × 10^9^/L, estimated glomerular filtration rate by Cockroft-Gault of ≥50 mL/minute, total serum bilirubin ≤1.5 × upper limit of normal, and fasting plasma glucose of <140 mg/dL (≤7.8 mmol/L), and platinum-refractory disease (defined as progression while receiving platinum-based therapy) was mandated in the dose expansion. There was no restriction on prior lines of systemic therapy, with the exception of prior PI3K inhibitors. Exclusion criteria included grade ≥2 peripheral neuropathy, grade ≥2 sensorineural hearing loss, recent radiotherapy or major surgery, uncontrolled brain metastases, or recent major cardiovascular or thrombotic event. Patients with known diabetes were eligible, if on stable noninsulin regimens and hemoglobin A1C of less than 6.4%. A more stringent hemoglobin A1C requirement of <7% for all patients was added in 2018.

This study was approved by the institutional review board at the University of California, San Francisco (NCT02620839) and followed the Declaration of Helsinki and Good Clinical Practice guidelines. All patients provided written informed consent.

### Study Design

This study was a phase Ib open label study of alpelisib in combination with cisplatin in patients with advanced solid tumor malignancies. Dose escalation followed standard 3+3 design (12). Two different weekly doses of cisplatin (30 and 35 mg/m^2^) were evaluated in combination with escalating doses of alpelisib, which was administered daily. A treatment cycle was defined as 21 days.

A dose-limiting toxicity (DLT) was defined as any grade ≥3 nonhematologic, treatment-related event, grade ≥3 febrile neutropenia, or grade 4 neutropenia, grade 3 thrombocytopenia with clinically significant bleeding or grade 4 thrombocytopenia, grade 3 hyperglycemia for >7 consecutive days despite oral antidiabetic treatment or grade 4 hyperglycemia. Patients who received less than 75% of the proposed dose for any reasons other than treatment-related toxicity in the first cycle were considered inevaluable for toxicity and were replaced. Patients had to have completed at least 75% of alpelisib doses and a minimum of two cisplatin infusions during the first cycle of treatment to be evaluable. As specified in the protocol, only evaluable patients were included in efficacy analyses, rather than an intention-to-treat analysis.

### Safety and Efficacy Assessments

Clinical and laboratory assessments were performed at baseline and once weekly prior to study drug administration during each cycle. Radiographic assessments were conducted every two cycles at the end of cycles 2, 4, and 6, and then every 3 cycles thereafter. Adverse events were graded using the Common Toxicity Criteria version 4.03.

### Study Objectives and Statistical Methods

The primary objective of this study was to determine the MTD and recommended phase II dose (RP2D) of alpelisib in combination with weekly cisplatin. Secondary objectives included determining the objective response rate (ORR), median progression-free survival (PFS), and characterizing the safety profile of the combination of alpelisib and cisplatin. The ORR was descriptively reported. Kaplan–Meier product limit method was performed to estimate the median PFS. The incidence and severity of adverse events were also descriptively reported. Stata version 15.1 was used for all statistical analyses.

### Data Availability Statement

The data generated in this study are not publicly available due to clinical details that could compromise patient privacy but are available upon reasonable request from the corresponding author.

## Results

### Study Population and Patient Disposition

Twenty-three patients were enrolled in this study between September 2016 to August 2018. Median age was 58 years (range 37–83), and 57% of patients were female. Baseline demographics and characteristics are detailed in Table 1. The majority of patients had >3 lines of prior therapy before study entry (91%) with a median prior therapy of 4 (range 1–10), with 18 (78%) patients with platinum-refractory disease (defined as progression while receiving prior platinum). Baseline median glycosylated hemoglobin A1C was 5.4%. Median duration of treatment for evaluable patients measured 17.86 weeks (range 6.3–40.7). Nine (39%) patients discontinued the study during cycle 1 without progression (*n* = 8 due to adverse event), but did not undergo staging studies in cycle 2. Of 14 evaluable patients, 11 patients demonstrated progressive disease, 1 patient discontinued due to toxicity, 1 patient was hospitalized for palliative care and later transitioned to hospice, and 1 patient withdrew from the study.

**TABLE 1:** Baseline patient characteristics of 23 patients enrolled in this phase Ib study

Baseline characteristics	Study cohort (*N* = 23)
Median age (range), years	58 (37–83)
Gender	
Male	10 (43%)
Female	13 (57%)
Race/ethnicity	
White	18 (78%)
Asian	4 (17%)
Hispanic	1 (4%)
ECOG performance status	
0	19 (83%)
1	4 (17%)
Tumor histology	
Prostate	5 (22%)
Cervical	3 (13%)
Endometrial	3 (13%)
Breast	2 (9%)
Anal	2 (9%)
Penile	2 (9%)
Other^a^	6 (26%)
Number of lines of prior therapy	
1	1 (4%)
2	1 (4%)
3	6 (26%)
4	8 (35%)
≥5	7 (30%)
Platinum-refractory disease (i.e., progression while receiving platinum therapy)	18 (78%)
Patient disposition	
Disease progression	11 (48%)
Adverse event	9 (39%)
Adverse event in first cycle	8 (35%)
Patient withdrawal	1 (4%)
Hospitalization for palliative care	1 (4%)
Death	1 (4%)

^a^Other cancers: angiosarcoma (*n* = 1), cholangiocarcinoma (*n* = 1), gastroesophageal junction (*n* = 1), ovarian (*n* = 1), rectal (*n* = 1), vaginal (*n* = 1).

### Determination of MTD and RP2D

Of the 23 patients enrolled across the four dose levels (Table 2), three patients experienced grade 4 hyperglycemia as a DLT: two at alpelisib 250 mg and one at alpelisib 300 mg. In addition to these cycle 1 DLTs, the post DLT period appeared intolerable for patients receiving alpelisib >250 mg and cisplatin >35 mg/m^2^. Given the challenges with delivering these higher doses, the MTD was alpelisib 250 mg/day on days 1–21, in combination with cisplatin 30 mg/m^2^ on days 1, 8, and 15 (cohort 2A). Two patients were treated at the MTD in the dose expansion cohort prior to study closure due to the toxicities of the combined adverse event profile and decision to pivot to a planned carboplatin and alpelisib combination trial.

**TABLE 2:** Dose-limiting toxicities and determination of MTD and RP2D

Alpelisib dose, mg/day on days 1–21	Cisplatin dose, mg/m^2^ days 1, 8, 15	Cohort	Number of patients	Number of DLTs	DLT Description
200	30	1A	4	0	
250	30	2A	7	1	Grade 4 hyperglycemia
250	35	2D	4	1	Grade 4 hyperglycemia
300	30	3C	6	1	Grade 4 hyperglycemia

### Safety Results

Treatment-related grade 3–4 toxicities included hyperglycemia (13%; Table 3), rash (13%), neutropenia (9%), anemia (4%), thrombocytopenia (4%), neuropathy (4%) and diarrhea (4%). The most frequent treatment-related adverse events of any grade included: fatigue (52%), diarrhea (39%), nausea (38%), hyperglycemia (30%), anemia (22%), nephropathy (17%), and thrombocytopenia (13%). Nine patients were removed from study due to adverse events. There were no treatment-related grade 5 adverse events. One patient died secondary to hypoxemic respiratory failure from a hospital-acquired pneumonia, which was not deemed related to the study drug. The other eight patients developed an AE during cycle 1, including: neuropathy, rash, hospitalizations for MRSA bacteremia, small bowel obstruction, and pneumonia, and 3 DLTs (all grade 4 hyperglycemia).

**TABLE 3:** Any grade and grade 3 adverse events and toxicities divided by cohort

Cohort	1A	2A	2D	3C	MTD
***N***	**4**	**7**	**4**	**6**	**2**
Grade 3					
Anemia	1	0	0	0	0
Neutropenia	1	1	0	0	0
Thrombocytopenia	0	0	0	0	1
Rash	0	0	1	1	1
Hyperglycemia	0	0	0	3	0
Hyponatremia	0	0	1	2	0
Pleural effusion	1	0	0	0	0
Diarrhea	0	1	0	0	0
Neuropathy	1	0	0	0	0
Any grade					
Anemia	1	2	0	2	0
Neutropenia	1	2	0	0	0
Thrombocytopenia	0	1	0	2	1
Rash	1	1	1	1	1
Hyperglycemia	1	1	1	4	0
Hyponatremia	1	0	1	2	0
Elevated creatinine	2	2	0	0	0
Fatigue	4	5	1	2	0
Nausea	2	5	1	1	0
Diarrhea	1	5	1	2	0

**FIGURE 1:**
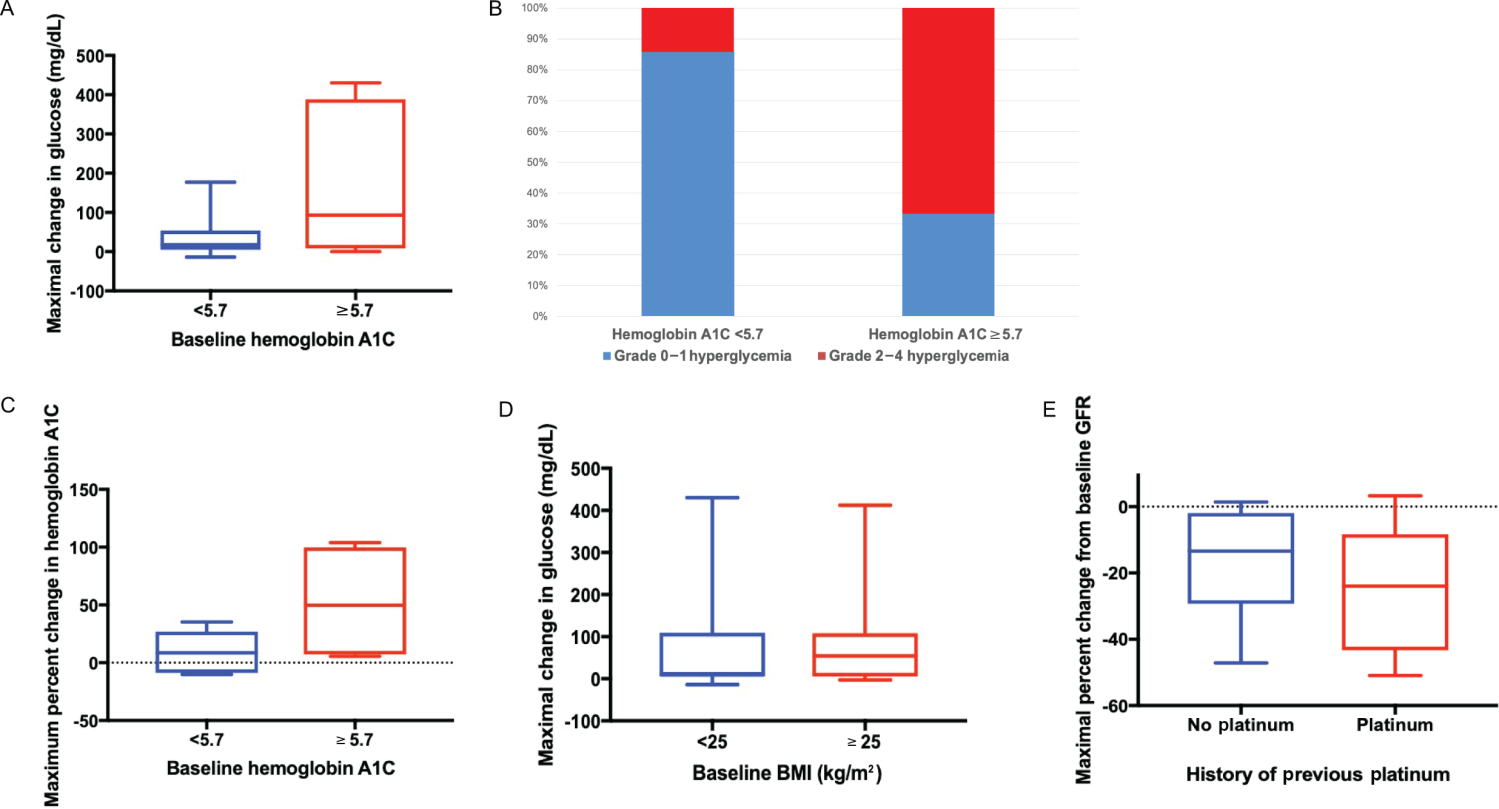
**A,** Maximal change from baseline fasting blood glucose (mg/dL). **B,** Percentage of patients with grades 0–1 and 2–4 hyperglycemia. **C,** Maximal change in hemoglobin A1C. **D,** Maximal change in BMI. **E,** Maximal percent change from baseline GFR based on history of previous platinum therapy.

Blood glucose and glycosylated hemoglobin A1C were obtained at baseline and then every 3 cycles. Hyperglycemia was seen among patients with both groups, those with normal (<5.7%) and elevated baseline hemoglobin A1C (≥ 5.7%). The effects were numerically more pronounced in the latter group, but this did not reach statistical significance (Mann—Whitney test, *P* = 0.07; Fig. 1A). A greater percentage of patients with a baseline hemoglobin A1C ≥5.7 demonstrated grade 2 hyperglycemia or higher (67% vs. 14%; *P* = 0.03, Fig. 1B). A rise in hemoglobin A1C in those with higher baseline hemoglobin A1C ≥5.7 (Mann—Whitney test, *P* = 0.21; Fig. 1C), appeared to be more marked. Baseline body mass index (BMI) did not influence hyperglycemia over the course of treatment (Mann—Whitney test, *P* = 0.61; Fig. 1D). All patients received dexamethasone during the administration of cisplatin.

**FIGURE 2:**
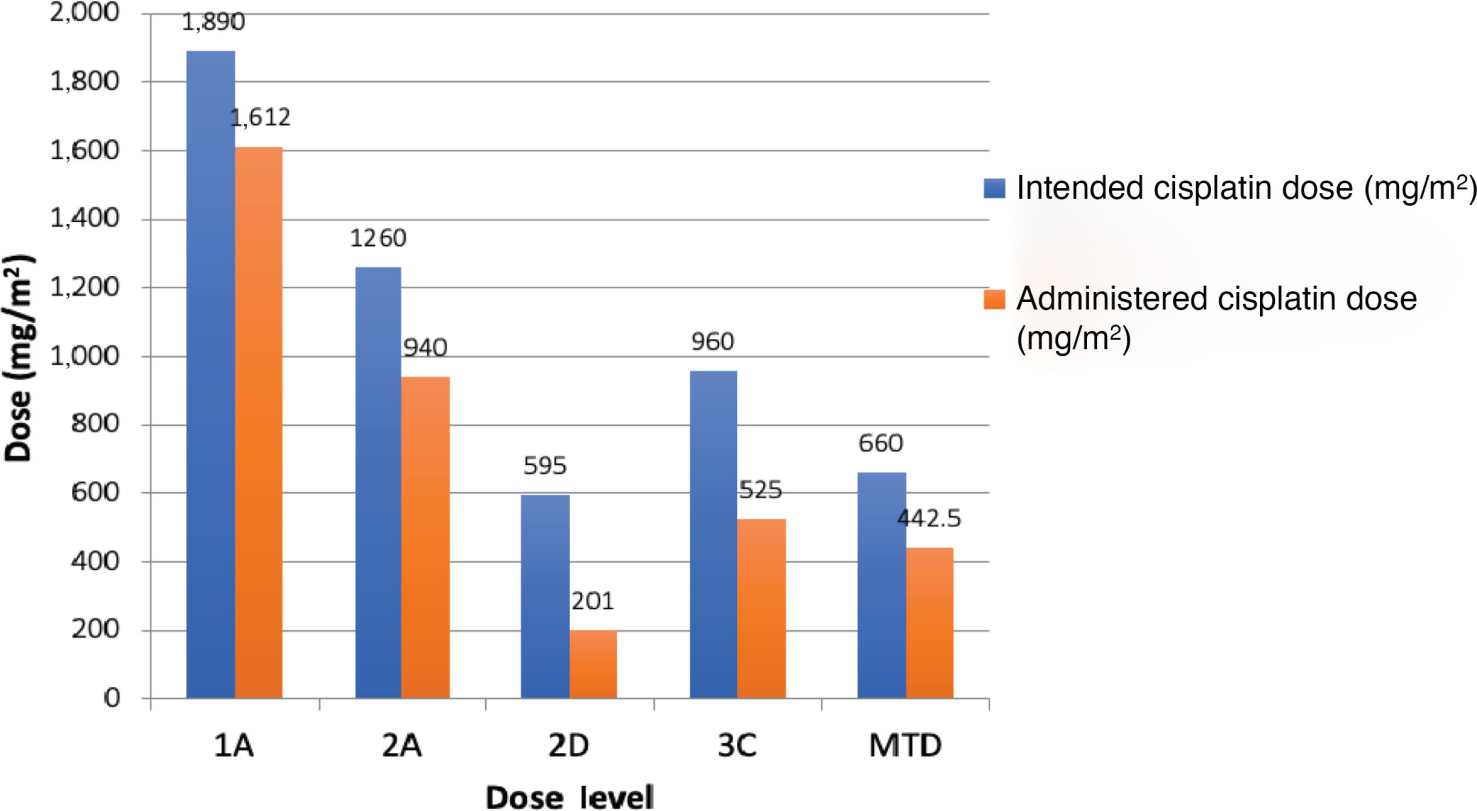
Dose intensity of cisplatin detailing intended and administered doses.

Study drug dose reductions and/or interruptions were related to platinum-induced nephrotoxicity (*n* = 10, 43%), hyperglycemia (*n* = 6; 26%), thrombocytopenia (*n* = 3; 13%), neutropenia (*n* = 2; 9%), and other (*n* = 8; 35%; Supplementary Table S1). Specifically, cisplatin dose changes and/or interruptions were necessitated for the following reasons: interruptions due to nephrotoxicity (17%), dose adjustment for nephrotoxicity (26%), adverse event other than nephrotoxicity (35%), and other reasons including weight loss, open wound requiring antibiotics, and concern for disease progression (22%). Nephrotoxicity was not more pronounced in patients who previously received platinum-based therapy (Mann—Whitney test, *P* = 0.35; Fig. 1E). There was also no difference in baseline GFR between patients who received cisplatin 35 mg/m^2^ compared with 30 mg/m^2^ (*P* = 0.94). The intended weekly cisplatin dose of 35 mg/m^2^ (Fig. 2) was poorly tolerated with considerable need for dose interruptions and dose modifications, resulting in delivery of only 33.8% of intended dose intensity in cohort 2D. In contrast, a dose intensity of 55%–85% could be reached with 30 mg/m^2^.

### Efficacy Analyses

**FIGURE 3:**
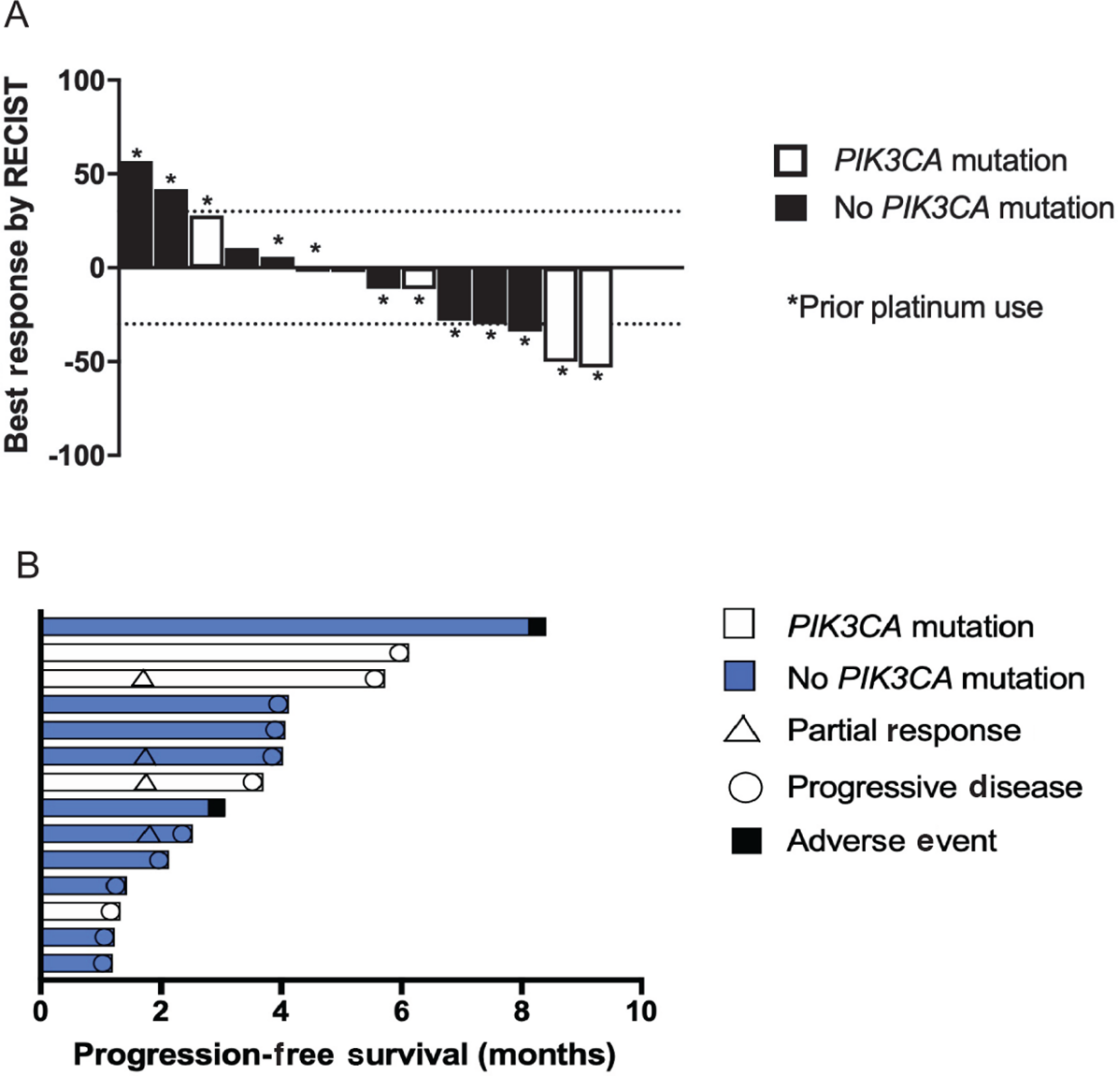
**A,** Waterfall plot demonstrating maximal change from baseline by RECIST 1.1 criteria in evaluable patients, **B,** Swimmer plot showing *PIK3CA* mutation status, progression-free survival, and best response by RECIST 1.1 criteria among evaluable patients.

Of the 23 patients, nine could not tolerate the combination to stay on trial through the first cycle, and 14 patients were evaluable for objective response. These evaluable patients received the following study treatment doses: alpelisib 200 mg with cisplatin 30 mg/m^2^ (*n* = 2), alpelisib 250 mg with cisplatin 30 mg/m^2^ (*n* = 7), alpelisib 250 with cisplatin 35 mg/m^2^ (*n* = 1), and alpelisib 300 mg with cisplatin 30 mg/m^2^ (*n* = 4). Four patients (29%) demonstrated a confirmed partial response, and 7 patients experienced disease stabilization (Fig. 3) for a disease control rate of 79%. Of the four responders, tumor sites included two endometrial cancers, cervical neuroendocrine carcinoma, and penile SCC. Patients received between 2 and 4 lines of prior therapy (50% with 4 prior lines), and all had previously progressed on platinum-based therapy (2 with platinum-refractory disease, 2 with platinum-resistant disease on first-line chemotherapy). Two of these four patients demonstrated a *PIK3CA* mutation. Overall median PFS measured 4.3 months (95% CI, 1.6–4.5), and the median duration of response for those with partial response and stable disease measured 21 weeks.

### Genomic Analysis

Twenty-one (91%) patients had somatic tumor profiling results available by standard gene panels. Five patients were found to have *PIK3CA* alterations (E545K in 2 patients, E726K, I311V, and Q546K), and no concurrent *KRAS* or *BRAF* mutations were identified. There was no significant difference in PFS when stratified by *PIK3CA* mutation status (*P* = 0.36). Recognizing the heterogeneity of the cohort and small sample size, further association between molecular alterations and clinical outcomes was not performed.

## Discussion

Given the high likelihood of platinum resistance, either *de novo* or acquired, much emphasis has been placed on finding novel agents to overcome resistance. Targeting the PI3K pathway has been proposed as a potential mechanism to overcome chemoresistance. In this study, we conducted a clinical trial using a combination of alpelisib and cisplatin in patients with advanced solid tumor malignancies who were heavily pretreated, with the intention of enrolling additional patients with HPV-associated tumors in the dose expansion cohort. The ORR for evaluable patients measured 29%; however, significant toxicity led to the discontinuation of 9 patients in the first cycle rendering a considerable number of patients inevaluable for response. Three of these patients experienced a DLT with grade 4 hyperglycemia (glucose >500 mg/dL) within cycle 1 of this combination regimen. Thus, potential benefits may have been obscured and the progression-free survival was relatively short. In addition, cumulative toxicity led to frequent treatment interruptions and further limited the ability for patients to stay on study. These therapeutic challenges prompted a decision to close the study and to reconsider an alternative platinum agent with lower nephrotoxicity rates and less concurrent steroid use. While it appears that there is a signal for efficacy with the combination of alpelisib and platinum therapy, the toxicities remained a barrier for prolonged treatment and full assessment of efficacy.

The ORR measured 29%, with all four patients having previously received platinum-based therapy suggesting the potential to reverse platinum resistance. Despite the limited sample size, this promising response rate highlights that this combination should be further explored as a mechanism to overcome platinum resistance, albeit with a different platinum backbone and close tissue-based glucose monitoring.

While all our patients received standard pre-medication with dexamethasone, we did not see a hyperglycemia rate higher than that used in the SOLAR-1 trial, which combined alpelisib with fulvestrant in patients with advanced *PIK3CA*-mutated hormone-positive breast cancer (2). However, hyperglycemia was pronounced and rapid in onset, which may have been further compounded by the required use of steroids given with cisplatin. In our analyses examining hyperglycemia, we subdivided hemoglobin A1C by clinical relevance, using a conservative hemoglobin A1C cut-off of 5.7%, which is the diagnostic threshold for pre-diabetes. The change from baseline fasting blood glucose was higher among those patients with a baseline hemoglobin A1C ≥5.7%. Given the pharmacologic management dictated by the protocol in the management of hyperglycemia, we did not see this translate into a significant increase in hemoglobin A1C over the course of study treatment in this subgroup of patients. Moving forward, all patients on alpelisib should undergo close glucose monitoring, particularly for those with a hemoglobin A1C ≥5.7%. Our group previously reported the impact of patient ethnicity on the metabolic implications of *PIK3CA* inhibitor treatment, with greater glycemic impact seen in Asian patients (13). However, with the small sample size, no observations could be drawn for the four Asian patients in our study.

To our knowledge, this is the first study of combination alpelisib and cisplatin among patients with advanced solid tumor malignancies. Day and colleagues recently reported a phase I trial of the same combination of alpelisib and cisplatin in the context of concurrent chemoradiation for 9 patients with locoregional advanced head and neck squamous cell carcinoma (14). In contrast to the weekly cisplatin schedule, we used in this study, chemoradiation consisted of cisplatin 100 mg/m^2^ every three weeks. The RP2D was alpelisib at 200 mg, and no DLTs were seen among the three DLT-evaluable patients at that dose. At the alpelisib 250 mg dose level, the two patients treated both experienced DLTs. Grade 2 alpelisib-related hyperglycemia was seen in one patient (at the 200 mg dose level). The higher rate of hyperglycemia seen in our cohort is likely related to dosing, as has been reported previously (15). We surmise that our RP2D of alpelisib was higher given that we used a weekly cisplatin dosing schedule without concurrent radiation. Despite the differences in patient population and intent of therapy, the results from both of our trials suggest that further study of alpelisib as a means to enhance platinum efficacy is warranted.

Among patients with metastatic head and neck squamous cell carcinoma, alpelisib has been studied in combination with cetuximab, where the 300 mg daily alpelisib dose was declared to be the RP2D (16). The phase II portion of the study comparing the combination with cetuximab alone has not yet been reported. With recent phase III data demonstrating the superiority of cisplatin over cetuximab in achieving locoregional control, the results for the alpelisib and cetuximab combination will be helpful in better understanding the synergistic clinical activity with concomitant PI3K and EGFR inhibition (9). This trial of alpelisib with cisplatin has demonstrated the safety and promising ORR of this combination, and the planned combination of alpelisib with carboplatin may be worth exploring further in the head and neck cancer subgroup.

In this heterogeneous small cohort, we did not find any specific associations with *PIK3CA* mutation status in this phase Ib trial. No concurrent *KRAS* or *BRAF* mutations were seen. While the link between *PI3KCA* alterations and alpelisib has been clearly established in hormone-positive breast cancer, many other clinical trials have not demonstrated such associations (2, 15, 17–20). This may be related to the complexity of the PI3K pathway with interactions with other signaling pathways, and larger studies regarding its role as a potential predictive biomarker in HPV-associated malignancies would be necessary (21).

Limitations of this study include the small sample size, lack of control group, and lack of patient data on single-agent treatment. Incorporating pre- and post-treatment biopsies would have been helpful in delineating the impact of the PI3K pathway in cisplatin resistance across various tumor types, as well as determining the optimal biologic dose. Due to the toxicity profile limiting prolonged treatment, this study was stopped prior to completion of the dose expansion phase, thus precluding investigation of the MTD in a larger prospective cohort. Furthermore, early closure prevented planned pharmacokinetic and pharmacodynamic analyses to better comprehend the impact of dose reductions and interruptions. These limitations will be addressed in a planned prospective clinical trial using carboplatin with alpelisib in a similar patient population. Carboplatin is much better tolerated compared to cisplatin with lower nephrotoxicity rates, and we plan to minimize concurrent steroid use to avoid exacerbation of hyperglycemia.

## Conclusion

In conclusion, the addition of alpelisib to cisplatin demonstrated responses among patients with solid tumor malignancies and platinum resistance, but toxicities limited time on treatment. To our knowledge, this is the first study of alpelisib and cisplatin in patients with advanced solid tumor malignancies. Future prospective studies are planned using carboplatin with alpelisib to improve the toxicity and tolerability.

## Supplementary Material

Supplementary Table S1 A) Rationale for study drug dose interruption or reduction; B) Rationale for cisplatin dose interruption or reduction; C) Cisplatin dose intensity, stratified by dose level
