# Supplementary material for: A Phase IB Trial of the PI3K Inhibitor Alpelisib and Weekly Cisplatin in Patients with Solid Tumor Malignancies
Source: Cancer Res Commun. 2022 Jul 1;2(7):570–6. doi: 10.1158/2767-9764.CRC-22-0028 (PMC10010328; doi:10.1158/2767-9764.CRC-22-0028)
Supplement: Supplementary Table S1 — A) Rationale for study drug dose interruption or reduction; B) Rationale for cisplatin dose interruption or reduction; C) Cisplatin dose intensity, stratified by dose level [file crc-22-0028-s01.docx]

**Supplemental Table 1.** A) Rationale for study drug dose interruption or reduction; B) Rationale for cisplatin dose interruption or reduction; C) Cisplatin dose intensity, stratified by dose level

A)

|  | **Nephrotoxicity** | **Neutropenia** | **Thrombocytopenia** | **Hyperglycemia** | **Other** |
| --- | --- | --- | --- | --- | --- |
| 1A | 2 | 1 | 0 | 1 | 3 |
| 2A | 3 | 1 | 0 | 1 | 1 |
| 2D | 2 | 0 | 0 | 1 | 0 |
| 3C | 2 | 0 | 2 | 3 | 4 |
| MTD | 1 | 0 | 1 | 0 | 0 |
| Total | 10 | 2 | 3 | 6 | 8 |

B)

|  | **Adverse event other than nephrotoxicity** | **Dose held for nephrotoxicity** | **Dose reduced for nephrotoxicity** | **Other** |
| --- | --- | --- | --- | --- |
| 1A | 1 | 0 | 3 | 1 |
| 2A | 2 | 2 | 1 | 1 |
| 2D | 0 | 2 | 1 | 0 |
| 3C | 3 | 0 | 1 | 3 |
| MTD | 2 | 0 | 0 | 0 |
| Total | 8 | 4 | 6 | 5 |

Other reasons for cisplatin dose interruption or reduction include weight loss requiring change in dosing, open wound requiring antibiotics and hold, and concern for disease progression.

C)

| **Dose Level** | **Number of Patients** | **Intended Alpelisib Dose (mg daily)** | **Intended Cisplatin Dose (mg/m^2^)** | **Intended Cisplatin Dose Delivery (mg/m^2^)** | **Administered Cisplatin Dose (mg/m^2^; % dose intensity)** |
| --- | --- | --- | --- | --- | --- |
| 1A | 4 | 200 | 30 | 1890 | 1612 (85.3%) |
| 2A | 7 | 250 | 30 | 1260 | 940 (74.6%) |
| 2D | 4 | 250 | 35 | 595 | 201 (33.8%) |
| 3C | 6 | 300 | 30 | 960 | 525 (54.7%) |
| MTD | 2 | 250 | 30 | 660 | 442.5 (67%) |
